# Perceived stress and allostatic load: Results from the *All of Us* Research Program

**DOI:** 10.1371/journal.pone.0330106

**Published:** 2025-08-08

**Authors:** Faith Morley, Lauren Mount, Anjile An, Erica Phillips, Rulla M. Tamimi, Kevin H. Kensler

**Affiliations:** 1 Department of Population Health Sciences, Weill Cornell Medicine, New York, New York, United States of America; 2 Sandra and Edward Meyer Cancer Center, Weill Cornell Medicine, New York, New York, United States of America; 3 Division of General Internal Medicine, NewYork-Presbyterian/Weill Cornell Medicine, New York, New York, United States of America; Northern Arizona University, UNITED STATES OF AMERICA

## Abstract

The rising prevalence of individuals reporting extreme stress has major public health implications as it increases vulnerability to accelerated premature biological aging, thus increasing risk of chronic disease. To examine the impact of stress on premature biological aging, we assessed the association between exposure to increased stress, quantified by the Perceived Stress Scale, and odds of high allostatic load (AL). To illuminate previously unexplored socio-contextual factors, we controlled for self-reported individual and neighborhood social determinants of health that included discrimination, loneliness, food insecurity, neighborhood disorder, and neighborhood social cohesion. We utilized a cross-sectional design to examine the association between perceived stress and AL among 7,415 participants ages 18–65 in the *All of Us* Research Program, who enrolled from 2017–2022. We used logistic regression to evaluate the association between stress and high AL, controlling for sociodemographic factors and self-reported social determinants of health. Participants who were younger, receiving Medicaid, or Hispanic had increased prevalence of high stress. High stress was associated with elevated odds of high AL in age and sex-adjusted models (OR=2.18, 95%CI = 1.78, 2.66, high stress vs. low), an association which remained significant after adjusting for social determinants of health (OR=1.29, 95%CI = 1.01, 1.65). Using restricted cubic splines, high stress was significantly associated with increased odds of high AL, even after controlling for upstream individual and neighborhood-level determinants of health. While individuals living below the medium poverty-to-income ratio demonstrated little appreciable association between high stress and increased odds of high allostatic load, those living above the median poverty-to-income ratio reporting increased stress appeared to have increased odds of high allostatic load. Through addressing the upstream factors causing undue burdens of stress, which particularly affect marginalized communities and younger generations, we can begin to address premature biological aging and the comorbid conditions it accompanies.

## Introduction

The prevalence of stress is increasing, with 24% of adults reporting high stress in 2023, up from 19% in 2019 [1]. Chronic stress is linked to numerous health conditions, including coronary heart disease, metabolic syndrome, hypertension, and diabetes [2]. As a result of the perpetuation of historical injustices and inequality, exposure to stress differs by many demographic, social, and environmental factors. While high stress is common across all racial and ethnic groups, it has been found non-Hispanic Black and Hispanic individuals report higher levels of stress than non-Hispanic White individuals [3]. Recent studies suggest Generation X, Generation Z, and the Millennial generation are unduly burdened by increasing stress, while individuals over 65 report decreasing levels [4,5]. The prevalence of high stress increased significantly from 2019 to 2023 across all age groups, except in adults over age 65 years [1]. Exposure to the same stressor can elicit responses of varying magnitudes across individuals, as the responses are dictated by inherent neurological capacity and self-regulation skills, which are profoundly shaped by lived experiences [6]. The linkage of exposure to the hormones related to stress responses and increased physiologic dysregulation, chronic disease, and premature death is substantiated by a robust body of empirical evidence. The pathogenesis of this purported biosocial pathway can be observed in a series of biological markers [7–10].

The conceptualization of allostatic load (AL) attempts to elucidate how stressors impact individuals’ health, by proposing a mechanism through which physiologic systems respond to stressors to maintain stability in the body [11]. Stress response hormones, such as cortisol or epinephrine, are the first to become elevated, resulting in subclinical dysregulation that impacts the cardiovascular, metabolic, and immune systems [11,12]. Ultimately, AL is the cumulative effect of the body’s effort to maintain this allostasis during stressful exposures that leads to ‘weathering’, the accumulation of the physiologic cost of chronic stress, and thus premature aging, a precursor to morbidity and mortality [10,12].

Individual exposure to stress is predicated on a dynamic interplay of interpersonal factors, such as daily life interactions, family and social dynamics, and isolation and social support, but also the environmental and social contexts in which we exist [13–17]. Factors like age, sex, and income can further modulate an individual’s level of perceived stress and individual stress response. To fully contextualize how individuals experience perceived stress and therefore stress response exposure, the impact of other upstream determinants of health must be considered. Disproportionate exposure to adverse neighborhood and social determinants can cause a compounding effect in relation to perceived stress levels, thus increasing risk of high AL [18].

Prior studies have examined AL and its relationships with stress and environmental factors, but are characterized by smaller sample sizes, a focus on a specific minoritized group, or lack the combination of biomarkers and self-reported measures of stress [19,20]. We leveraged the multimodal data of the *All of Us* Research Program to examine how stress is associated with AL in the context of upstream social determinants of health and further examined heterogeneity in the association by age, sex, and income. We posit adjusting for exposures to disadvantage and deprivation will partially attenuate the association between high stress and odds of increased allostatic load. This study can provide further insight as to how stressors in an individual’s environment can impact the body’s stress response, ultimately leading to premature aging.

## Methods

### Study population

The *All of Us* Research Program is a longitudinal cohort initiative created by the National Institute of Health to create a comprehensive dataset of electronic health record (EHR) data, survey data, physical measurements, genomic data, and biospecimens, with emphasis on recruiting populations historically underrepresented in biomedical research. All community-dwelling individuals over the age of 18 living in the United States are eligible to enroll [21]. Launched in May 2017, there are 409,420 participants in the Controlled Tier V7 data (initially released April 20, 2023), with follow up through July 1, 2022. *All of Us* participants provide electronic consent upon enrollment. Participants can opt to link their EHR data from ~67 sites across the United States, with EHR data harmonized using the Observational Medical Outcomes Partnership (OMOP) common data model. Participants can also complete an in-person study visit during which program staff obtain physical measurements including heart rate, systolic and diastolic blood pressure, waist and hip measurements. All participants complete a survey on demographics and socioeconomic status, while completion of other surveys on topics such as overall health and lifestyle is optional. This study was deemed exempt by the Weill Cornell Medicine IRB committee (23-0726250). *All of Us* Research Program data are publicly available in a de-identified format through the Researcher Workbench.

### Perceived stress

To quantify our exposure of interest, self-perceived stress was measured using survey responses that make up Cohen’s Perceived Stress Scale (PSS) [22]. This scale more accurately measures the level of self-appraised stress from individual experiences, rather than quantifying the number of adverse events within a temporal constraint, as the physiologic response and magnitude of said response is dictated by the extent to which the individual views it as a threat, not the event itself [22]. This 14-item Likert-scale was scored according to the prescribed cut points (0–13 low, 14–26 moderate, and 27–40 high) [22].

### Allostatic load

There is great heterogeneity in AL scoring algorithms, with no consensus in selecting biomarkers [23]. We identified 12 components of AL covering seven physiologic systems (S1 Table). Our algorithm was informed by literature and the frequency with which measurements appear in the *All of Us* EHR data [2,19,24]. Of the 12 AL score components, 11 were measured biomarkers from the cardiovascular (systolic blood pressure, diastolic blood pressure, and heart rate), lipid metabolism (high-density lipoprotein [HDL] cholesterol, total cholesterol, and triglycerides), glucose metabolism (glucose), renal (creatinine), immune (albumin, white blood cell count, and the presence of an asthma diagnosis), and anthropometric systems (waist-to-hip ratio). Trained staff collect participants’ systolic and diastolic blood pressure, heart rate, and waist and hip measurements upon enrollment during an initial visit. Following the guidance of previous studies, we adjusted for the potential obfuscating effects of medication usage on cardiovascular, lipid metabolism, and glucose metabolism systems. Participants with record of medication for hypertension, diabetes, and hyperlipidemia were assigned a to the high-risk quartile for the relevant system; participants using antihypertensives assigned to the high risk quartile for systolic blood pressure, those using of lipid modifiers were assigned to the high risk quartile for LDL cholesterol, and those using diabetes medication were assigned to the high risk quartile for glucose, regardless of where their measured value fell. Medication records were collected through the EHR by querying RXNORM codes (S2 Table) [25].

In line with prior studies, participants were assigned a point for each component measure of AL in the highest-risk quartile (>75^th^ percentile for all AL components except for HDL and albumin, for which <25^th^ percentile was considered highest-risk) [26,27]. Quartiles were determined using cohort-wide distributions, apart from HDL, waist-to-hip ratio, and creatinine, for which sex-specific thresholds are used in clinical practice, and thus sex-specific quartiles were implemented [28,29]. A proxy for dysregulation of the immune system, a point was assigned for self-report in a survey or an EHR record of an asthma diagnosis [7,30,31]. The summation of these components is the AL score (range 0–12), which was transformed into a dichotomous outcome. ‘High AL’ was assigned to participants whose individual allostatic load sum fell above the 75^th^ percentile of the cohort’s scores (allostatic load score ≥6), while the remaining participants were classified as ‘Low AL’ (<6) [32].

For participants with multiple measures of the same biomarker in their EHR, the measurement nearest to the date of enrollment was selected [33,34]. Apart from medication usage and asthma diagnosis, all components of AL were required to be measured within a 3-year window, as to ensure adequate capture of participant health at the time of reporting of perceived stress.

### Covariates

Social determinants of health in *All of Us* are measured using an optional survey comprised of several social determinants, completed by 117,783 participants (29% completion rate) [21]. To control for upstream causes of perceived stress, self-reported individual- and neighborhood-level determinants of health were considered in our analyses. These included the Neighborhood Social Cohesion Scale (NSCS) [35], Neighborhood Disorder Scale (NDS) [36], the UCLA-6 Item Loneliness Scale (LON) [37], Everyday Discrimination Scale (EDS) [38], and a subset of Children’s HealthWatch Hunger Vital Sign (HVS) [39]. Quartiles were calculated for each scale, except for HVS, for which an affirmative answer to either item was categorized as food insecure [39]. For NDS, LON, and EDS, higher scores indicate higher levels of disorder, loneliness, and discrimination, respectively, while high NCSC scores indicate lower levels of social cohesion [35].

Other sociodemographic characteristics of interest included age, sex at birth, race and ethnicity, nativity, employment, education, marital status, poverty-income-ratio (PIR), smoking status, electronic cigarette usage, alcohol consumption (using the AUDIT-C screening tool) [40]. Separate self-reported race and ethnicity variables were combined to create a singular race and ethnicity variable, with categories in line with Office of Management and Budget standards [41]. Participants who identified as non-Hispanic Asian or Pacific Islander or non-Hispanic Middle Eastern and North African ultimately were included in the “other” category due to small sample sizes, as were participants who identified with multiple racial groups. Participants who did not report ethnicity or race were categorized as unknown. The PIR was calculated with yearly household income, using the midpoint of each reported income group divided by the participant’s federally determined poverty level (FPL) as determined by the participant’s reported family size. PIR was then transformed into a categorical variable of rounded tertiles, grouped as “<200%”, “200-400%”, and “>400%” of the FPL [10,42–44].

### Inclusion/exclusion criteria

**Fig 1:**
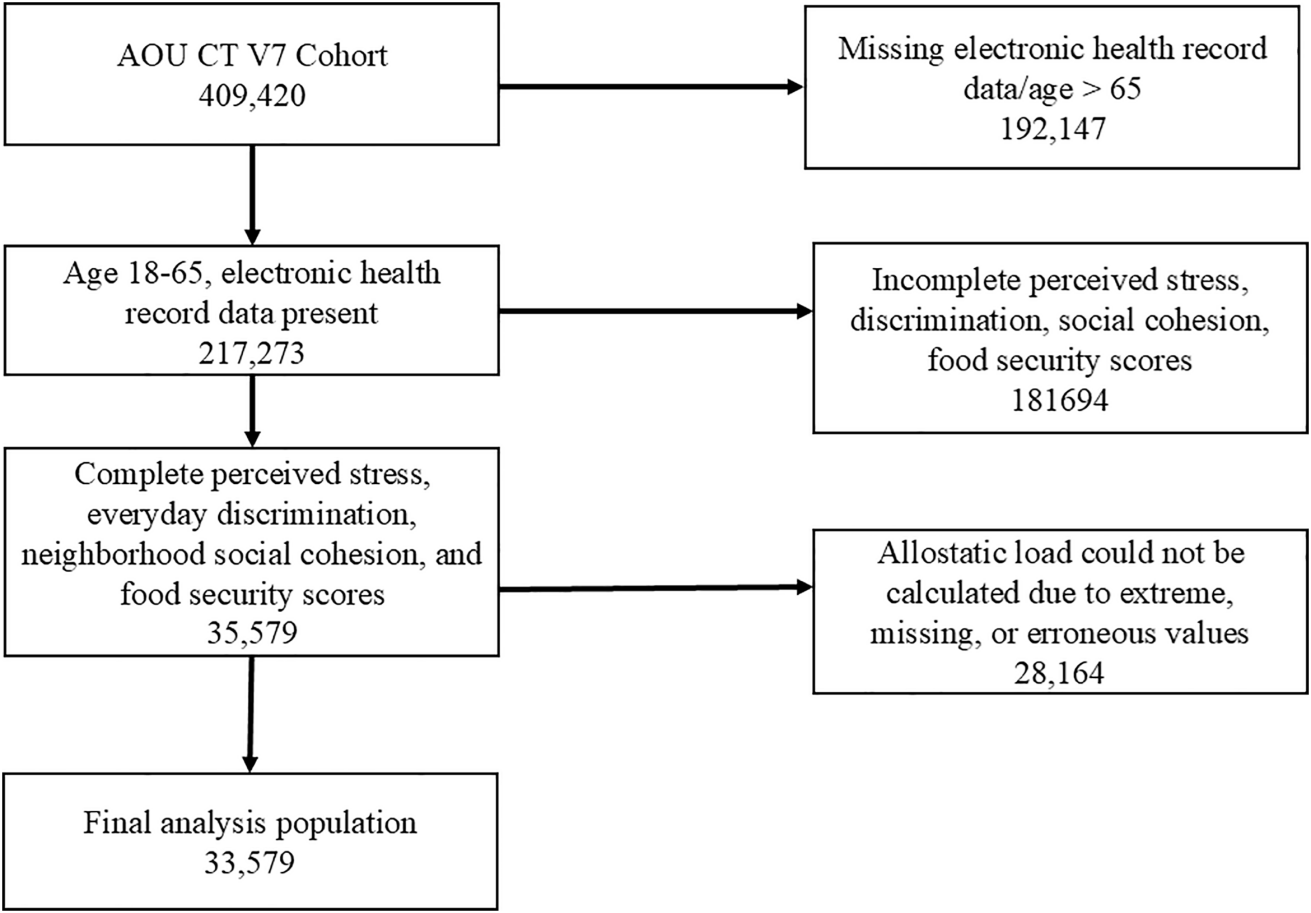
Exclusion flowchart. Flowchart for the identification of analytic population in All of Us Research Program Controlled Tier V7 data release.

Steps to identify the analytic population are summarized in Fig 1. We identified a cohort using the Controlled Tier V7 data release who consented to share their EHR data and had at least one recorded measurement for each of the 11 biomarker components of the AL algorithm. Participants with incomplete PSS, EDS, NSCS, LON, HVS, and NDS scales, or AL components not all measured within a 3-year window were excluded. Participants aged 65 or greater were excluded from analysis, as AL plateaus later in life and remains constant [45]. After excluding those without the requisite 11 biomarkers drawn no more than 3 years apart and complete scale scores and were under age 65, 7,415 participants met eligibility criteria (Fig 1).

### Statistical analyses

Seven of the biomarker components of our AL algorithm were ascertained from the EHR and were measured for clinical care and not research purposes. To account from non-random sampling for the availability of these biomarkers we used inverse probability weighting (S1 File). To examine the association between PSS as a categorical variable and AL while controlling for upstream factors, four sequentially-adjusted weighted logistic regression models were implemented. Model 1 adjusted for age and sex at birth. Model 2 controlled for education, race and ethnicity, nativity, employment, PIR category, and marital status [46]. Model 3, considered the primary model, further controlled for NDS, NSCS, LON, HVS, and EDS. Model 4 controlled for smoking status, alcohol consumption, and electronic cigarette usage, which were considered possible mediators on the causal pathway between stress and AL [47].

PSS was also modeled using restricted cubic splines to achieve greater flexibility to evaluate the association of perceived stress and high AL for the four sequentially-adjusted models. The appropriate number of knots was identified using Akaike Information Criterion (AIC), and ultimately three knots, placed at the 10^th^, 50^th^, and 90^th^ percentiles, were included. Finally, heterogeneity in the association between perceived stress and AL was evaluated by age, PIR, and sex at birth through incorporation of interaction terms in Model 3 (again with three knots). For evaluating heterogeneity, age was grouped by tertile, and PIR was dichotomized at the median. Odds ratios were plotted relative to the median perceived stress score of the modal category for each respective interaction, the significance of which was then determined by likelihood ratio test (15). Heterogeneity by race and ethnicity was not evaluated due to the limited sample sizes of Hispanic and non-Hispanic Black participants. We additionally performed a sensitivity analysis restricted to participants for whom all AL components were measured within a two-year window [26]. In line with *All of Us* Research Program requirements, all cells with <20 participants are suppressed. All statistical tests were two-sided with α = 0.05. All analyses were performed using R (Version 4.4.0) in the *All of Us* Research Program Researcher Workbench.

## Results

**Fig 2:**
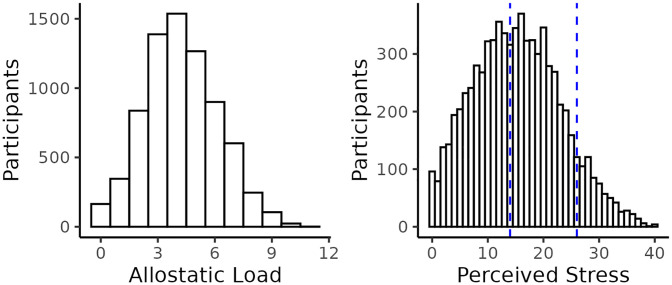
Distribution of allostatic load scores (left) and perceived stress scores (right) in the study population. Perceived stress scale groupings are represented by blue dashed lines (0-13 low, 14-26 moderate, and 27-40 high).

A total of 7,415 participants met eligibility criteria with median age 52.4 years at time of enrollment. 3,213 (43.3%) participants reported low stress, 3,566 (48.1%) reported moderate stress, and 636 (8.6%) reported high stress (Table 1; Fig 2). The median stress score was 15 overall but was inversely associated with age (18 for 18–39-year-old, 16 for 40–54-year-old, and 12 for 55–64-year-old participants). The cohort was predominantly composed of individuals who identified as non-Hispanic White (73.5%), followed by non-Hispanic Black (9.9%), Hispanic (8.8%), another race or ethnicity (4.4%), and unknown (3.3%). Participants that were female, who never married, Hispanic, unable to work, or lower PIR appeared to have nominally higher perceived stress. High levels of neighborhood disorder, discrimination, loneliness, and low levels of neighborhood social cohesion also appeared to report nominally increased levels of stress.

**Table 1:** *All of Us* Research Program participant characteristics^1^ by category of self-reported perceived stress (0-13 low, 14-26 moderate, and 27-40 high).

Characteristic	Low Stress N = 3213	Moderate Stress N = 3566	High Stress N = 636	Total N = 7415
**Allostatic Load**				
Low AL (<6)	2488 (77.4%)	2632 (73.8%)	418 (65.7%)	5538 (74.7%)
High AL (≥6)	725 (22.6%)	934 (26.2%)	218 (34.3%)	1877 (25.3%)
**Age Group**				
Under 40	448 (13.9%)	977 (27.4%)	241 (37.9%)	1666 (22.5%)
40–54	1039 (32.3%)	1357 (38.1%)	240 (37.7%)	2636 (35.5%)
55 and above	1726 (53.7%)	1232 (34.5%)	155 (24.4%)	3113 (42.0%)
**Sex at Birth**				
Female	2105 (65.5%)	2631 (73.8%)	476 (74.8%)	5212 (70.3%)
Male	1051 (32.7%)	<850	<150	<2050
Unknown	57 (1.8%)	<90	<20	<160
**Race and Ethnicity**				
NHW	2422 (75.4%)	2580 (72.3%)	447 (70.3%)	5449 (73.5%)
NHB	333 (10.4%)	344 (9.6%)	60 (9.4%)	737 (9.9%)
Hispanic	235 (7.3%)	354 (9.9%)	65 (10.2%)	654 (8.8%)
Other	129 (4.0%)	165 (4.6%)	34 (5.3%)	328 (4.4%)
Unknown	94 (2.9%)	123 (3.4%)	30 (4.7%)	247 (3.3%)
**Health Insurance**				
Private	2433 (75.7%)	2315 (64.9%)	297 (46.7%)	5045 (68.0%)
Medicare	154 (4.8%)	191 (5.4%)	38 (6.0%)	383 (5.2%)
Medicaid	311 (9.7%)	<690	<230	<1230
VA/Military	97 (3.0%)	<90	<20	<210
None	218 (6.8%)	286 (8.0%)	59 (9.3%)	563 (7.6%)
**Relationship Status**				
Domestic Partner	2134 (66.4%)	2030 (56.9%)	275 (43.2%)	4439 (59.9%)
No Longer Married	533 (16.6%)	656 (18.4%)	135 (21.2%)	1324 (17.9%)
Never Married	483 (15.0%)	781 (21.9%)	206 (32.4%)	1470 (19.8%)
Unknown	63 (2.0%)	99 (2.8%)	20 (3.1%)	182 (2.5%)
**Employment Type**				
Employed	2127 (66.2%)	<2200	<290	<4610
Retired	403 (12.5%)	<210	<20	<630
Unable	285 (8.9%)	495 (13.9%)	169 (26.6%)	949 (12.8%)
Unemployed	321 (10.0%)	568 (15.9%)	149 (23.4%)	1038 (14.0%)
Unknown	77 (2.4%)	105 (2.9%)	21 (3.3%)	203 (2.7%)
**Education**				
HS or less	328 (10.2%)	509 (14.3%)	105 (16.5%)	942 (12.7%)
Some College	680 (21.2%)	856 (24.0%)	201 (31.6%)	1737 (23.4%)
College	967 (30.1%)	1050 (29.4%)	178 (28.0%)	2195 (29.6%)
Advanced	1169 (36.4%)	<1060	<150	<2370
Unknown	69 (2.1%)	<100	<20	<190
**Birthplace**				
USA	2853 (88.8%)	3096 (86.8%)	577 (90.7%)	6526 (88.0%)
Outside USA	307 (9.6%)	<400	<50	<760
Unknown	53 (1.6%)	<80	<20	<150
**PIR Category**				
<200%	559 (17.4%)	1007 (28.2%)	293 (46.1%)	1859 (25.1%)
200-400%	568 (17.7%)	736 (20.6%)	134 (21.1%)	1438 (19.4%)
>400%	1751 (54.5%)	1430 (40.1%)	142 (22.3%)	3323 (44.8%)
Unknown	335 (10.4%)	393 (11.0%)	67 (10.5%)	795 (10.7%)
**Loneliness Quartile**				
Quartile 1	1543 (48.0%)	670 (18.8%)	40 (6.3%)	2253 (30.4%)
Quartile 2	810 (25.2%)	788 (22.1%)	61 (9.6%)	1659 (22.4%)
Quartile 3	616 (19.2%)	1110 (31.1%)	150 (23.6%)	1876 (25.3%)
Quartile 4	244 (7.6%)	998 (28.0%)	385 (60.5%)	1627 (21.9%)
**Everyday Discrimination Quartile**				
Quartile 1	1299 (40.4%)	622 (17.4%)	37 (5.8%)	1958 (26.4%)
Quartile 2	960 (29.9%)	821 (23.0%)	81 (12.7%)	1862 (25.1%)
Quartile 3	641 (20.0%)	1026 (28.8%)	125 (19.7%)	1792 (24.2%)
Quartile 4	313 (9.7%)	1097 (30.8%)	393 (61.8%)	1803 (24.3%)
**Food Security**				
Secure	3095 (96.3%)	3019 (84.7%)	426 (67.0%)	6540 (88.2%)
Insecure	118 (3.7%)	547 (15.3%)	210 (33.0%)	875 (11.8%)
**AUDIT C**				
Not Hazardous	1864 (58.0%)	2058 (57.7%)	399 (62.7%)	4321 (58.3%)
Hazardous	1151 (35.8%)	1221 (34.2%)	186 (29.2%)	2558 (34.5%)
Unknown	198 (6.2%)	287 (8.0%)	51 (8.0%)	536 (7.2%)
**Vaping History**				
No	2893 (90.0%)	2955 (82.9%)	454 (71.4%)	6302 (85.0%)
Yes	<310	<600	<200	<1120
Unknown	<20	<50	<20	<50
**Smoking History**				
Never Smoker	2176 (67.7%)	2285 (64.1%)	369 (58.0%)	4830 (65.1%)
Current	212 (6.6%)	350 (9.8%)	106 (16.7%)	668 (9.0%)
Former smoker	792 (24.6%)	<860	<170	<1820
Skip	33 (1.0%)	<90	<20	<140
**Neighborhood Social Cohesion Quartile**				
Quartile 1	1130 (35.2%)	732 (20.5%)	69 (10.8%)	1931 (26.0%)
Quartile 2	1143 (35.6%)	1178 (33.0%)	150 (23.6%)	2471 (33.3%)
Quartile 3	580 (18.1%)	883 (24.8%)	156 (24.5%)	1619 (21.8%)
Quartile 4	360 (11.2%)	773 (21.7%)	261 (41.0%)	1394 (18.8%)
**Neighborhood Disorder Quartile**				
Quartile 1	1225 (38.1%)	739 (20.7%)	91 (14.3%)	2055 (27.7%)
Quartile 2	840 (26.1%)	937 (26.3%)	156 (24.5%)	1933 (26.1%)
Quartile 3	667 (20.8%)	907 (25.4%)	156 (24.5%)	1730 (23.3%)
Quartile 4	481 (15.0%)	983 (27.6%)	233 (36.6%)	1697 (22.9%)

^1^Cells with <20 participants are suppressed in accordance with All of Us Research Program’s data dissemination policy.

Odds ratios for the association between categorical stress and high AL are shown in Table 2. In Model 1, adjusting for age and sex, individuals that reported high stress had increased odds of high AL (OR=2.18, 95% CI = 1.78, 2.66, high stress vs. low stress), while those who reported moderate stress had 28% higher odds (OR=1.28, 95% CI = 1.11, 1.42, moderate stress vs. low stress). When adjusting for sociodemographic characteristics (Model 2), the association attenuated but high stress continued to be associated with high AL (OR= 1.46, 95% CI = 1.17, 1.81). The association further attenuated in Model 3, which controlled for individual- and neighborhood-level determinants of health (OR= 1.28 for high vs low, 95% CI = 1.00, 1.63), with similar findings in Model 4, which accounted for potential behavioral mediators of the effect of stress on AL (OR=1.29 for high vs low, 95% CI = 1.01, 1.65). These findings were similar when restricting to participants with all AL components measured in a two-year window (S4 Table).

**Table 2:** Weight rated odds ratios (OR) and 95% confidence intervals (95% CI) for the association between perceived stress and high allostatic load.

	Model 1		Model 2		Model 3		Model 4	
Stress	OR	95% CI	OR	95% CI	OR	95% CI	OR	95% CI
Low	—	—	—	—	—	—	—	—
Moderate	1.28	1.14, 1.44	1.10	0.98, 1.25	1.02	0.89, 1.17	1.03	0.90, 1.19
High	2.18	1.78, 2.66	1.46	1.17, 1.81	1.28	1.00, 1.63	1.29	1.01, 1.65

Model 1 incorporates inverse probability of selection weights and includes age, sex at birth.

Model 2 includes for all variables in Model 1 plus education, race and ethnicity, health insurance, nativity, employment, poverty-to-income ratio group, and marital status.

Model 3 includes for all variables in Model 2 plus neighborhood disorder scale, neighborhood social cohesion scale, UCLA-Loneliness scale, everyday discrimination scale, and food security scale.

Model 4 includes for all variables in Model 3 plus smoking, alcohol consumption, and electronic cigarette use.

**Fig 3:**
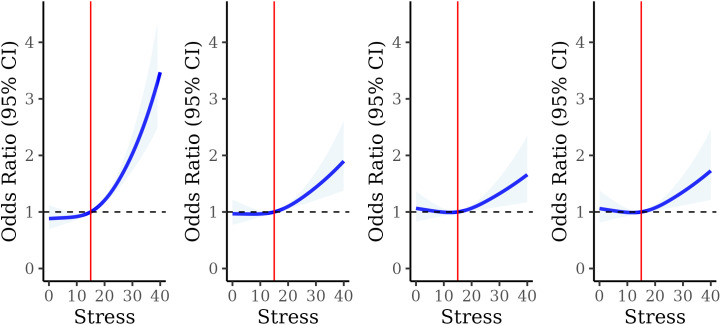
Odds ratios and 95% confidence intervals (95% CI) for the association between perceived stress score (PSS) and high AL using Model 1, Model 2, Model 3, and Model 4 (sequentially, left to right). PSS is modeled using restricted cubic splines. Model 1 includes age, sex at birth and IPW weights (p for nonlinearity = 0.003). Model 2 includes all variables in Model 1 plus educational attainment, race and ethnicity, health insurance, nativity, employment status, PIR, and marital status. Model 3 *includes* all variables in Model 2 plus neighborhood disorder, neighborhood social cohesion, loneliness, everyday discrimination, and food insecurity. Model 4 includes all variables in Model 3 plus cigarette smoking, electronic cigarette use, and alcohol consumption. Odds ratios are calculated relative to the median stress score, 15.

Fig 3 shows the odds ratios for high AL in relation to continuous stress modeled using restricted cubic splines. In the age- and sex-adjusted model (Model 1), higher levels of stress were strongly associated with high AL, however, as observed categorically, there was substantial attenuation of the association after controlling for socioeconomic and demographic characteristics (Model 2). Upon controlling for individual- and neighborhood-level determinants (Model 3), the association attenuated further but remained significant. Ultimately, adjustment for hypothesized causal behavioral mediators had little influence on the association between perceived stress and high AL (**Model 4**).

**Fig 4:**
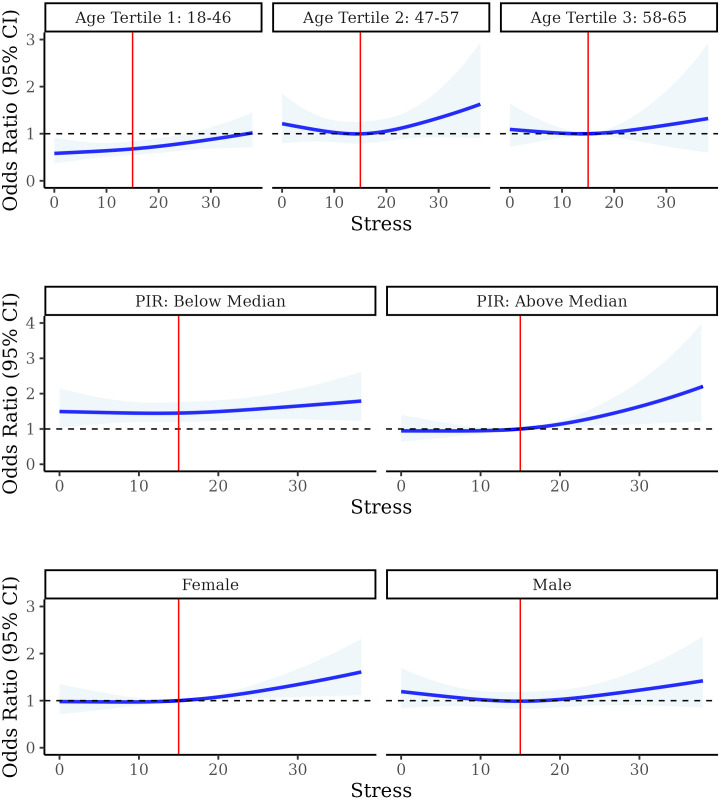
Odds ratios and 95% confidence intervals (95% CI) from restricted cubic splines for the association between perceived stress (PSS) and high AL. Stratified by tertile of participant age (p-heterogeneity = 0.62, referent group: Tertile 3)(top), poverty-to-income ratio (PIR, p-heterogeneity = 0.12, referent group: above median) (middle), and sex assigned at birth (p-heterogeneity = 0.81, referent group: female group) (bottom). Odds ratios are adjusted for age, sex at birth, PIR, educational attainment, race and ethnicity, health insurance, nativity, employment status, marital status, neighborhood disorder, neighborhood social cohesion, loneliness, everyday discrimination, and food insecurity.

The association between stress and high AL stratified by age, sex, and PIR is shown in Fig 4. The association between stress and high AL demonstrated similar associations between age tertiles. Despite the highest tertile of age having elevated odds of high AL than the lowest tertile, stress was similarly positively associated with higher AL across all adults, with the strongest association observed in those under 46 (p-heterogeneity = 0.62). Suggestive heterogeneity was observed between those living above and below the median PIR (p-heterogeneity = 0.12). Individuals living below the median PIR had increased odds of high AL than those living above the median PIR at baseline, but perceived stress was not associated with high AL in this population. However, for those living above the median PIR, increased levels of stress were suggestive of increased odds of high AL. The association between perceived stress and high AL did not differ by sex at birth (p-heterogeneity = 0.81).

## Discussion

We assessed the association between perceived stress and AL in the *All of Us* Research Program while adjusting for other upstream socioeconomic, environmental, and social factors. Our findings demonstrate that, in this cohort, higher levels of perceived stress are associated with increased odds of high AL. The association attenuates but was still observed when controlling for neighborhood, social, and environmental measures. When examining heterogeneity in the associations between stress and odds of high AL, increased levels of stress demonstrated a suggestively stronger association with increased odds of high AL in participants living above the median PIR, but did not differ by age nor sex assigned at birth.

In our sequentially fit multivariable-adjusted models, our findings are in line with prior studies of stress and AL, which observed that higher levels of education and wealth, and strong social support at home are associated with lower odds of high AL, while high perceived stress is associated with increased odds of high AL [9,10,48,49]. Our findings replicate and build upon these observations by demonstrating the association between stress and AL can be in part attributed to confounding by upstream determinants of health.

The homogeneity in the association between stress and AL by age has major public health implications. Previous studies suggest that older individuals benefit from learned resilience and coping skills [3,50,51]. Adults over 65 report lower levels of perceived stress and increased or steady levels of emotional well-being, while younger adults, especially those under 44, report increased stress [1]. This divide in perceived stress levels and coping becomes particularly evident when comparing the increasing prevalence of stress in all age groups except adults over 65 from 2019 to 2023 [1,3]. As AL has been described to increase sharply before the age of 60, after which it becomes somewhat constant, the increasing prevalence of stress in younger and middle-aged adults is particularly troublesome, as it is occurring arguably during the same time they are most sensitive to ‘weathering’ [1,45]. In our examination of heterogeneity by age-grouping, participants aged 18–46 demonstrated an almost linear increasing association between stress and odds of high AL, and participants aged 47–57 exposed to above average stress had increased odds of high allostatic load, while the oldest adults in our study, 58–65 demonstrated little increased risk. The suggested heterogeneity in these findings is particularly concerning when one considers adults aged 18–46 reported the highest median levels of perceived stress and adults aged 58–65 reported the lowest. Increased stress among younger adults and the consistent association between stress and AL across all adults, may portend that current young adults will exhibit greater physiologic dysregulation as they age [3]. Thus this age cohort may face increased risk of accelerated biologic aging, which has been attributed to higher rates of chronic conditions [1].

Extant literature pertaining to health outcomes supports our findings concerning heterogeneity in the association between perceived stress and odds of high allostatic load by PIR group. Previous studies have elucidated heterogeneity by socioeconomic status when evaluating the associations between other social and neighborhood determinants and health outcomes, finding that poverty acts as an accelerator upon biological aging [52]. Younger individuals living in poverty experience similar biological risks to their health as wealthier individuals two decades older [52]. This premature biological aging results in disparate mortality rates by socioeconomic status of such great proportions, peaking in their 50’s, that by old age there are no longer health status differentials, as people with the highest risk have died at younger ages (i.e., survivorship bias) [52]. We found participants below the median PIR had increased odds of high AL overall; however, we observed little appreciable increase in AL as stress increased in this group. Conversely, above the median PIR, higher stress was associated with increased odds of high AL. Individuals with lower PIR are more likely to be exposed to multiple adverse health determinants and increased biological risk factors, and thus the singular impact of one of these determinants on AL is likely to be less pronounced. Accordingly, heightened stress may not impact AL among individuals with lower PIR to the extent it does in individuals with greater levels of wealth, suggesting inherent inequity between groups, rather than diminished risk from high AL among those with lower PIR [53].

A strength of this work is the unique combination of EHR, physical measurements, and a robust collection of self-reported individual and neighborhood determinants of health that allow for the analysis of multilevel determinants of health in a novel way. This study builds upon prior studies by accounting for a broad set of participant-reported multilevel determinants [20,34,46]. Our study also has some notable limitations. Similar to many previous retrospective AL studies, some of the component biomarkers were measured in the context of clinical care and not for research purposes. Therefore, were unable to operationalize stress-related hormones in our algorithm, such as cortisol, DHEA-S, or epinephrine, due to limited availability in the EHR [27]. We measured AL over a 3-year window and the component biomarkers can naturally change over this period. However, our findings were robust when restricting to a narrower time window. Furthermore, a condition for inclusion in this study was a combination of complete PSS, EDS, NSCS, LON, HVS, and NDS scores, which are part of a survey completed by only 29% of the participants, who have greater levels of wealth, education, and employment than the overall cohort [21]. However, the distribution of perceived stress within our analytic population was similar to prior studies.

In summary, we found that perceived stress is associated with increased odds of high AL, confirming associations found in prior studies. However, by controlling for multiple self-reported individual and neighborhood factors not previously done, the results of this study provide a nuanced understanding of the association, emphasizing the significant contributions of environmental and social factors in the context of physiologic dysregulation. In the context of the rising prevalence of stress among younger individuals, our findings highlight the increased risk of physiologic dysregulation facing younger members of our society. As this makes younger generations vulnerable to increased risk of accelerated biologic aging and ensuing chronic conditions, this critical issue calls for further research and new policies to provide resources to younger individuals to decrease the prevalence of stress and increase coping methods. These results also reemphasize the weight of systemic inequities when addressing risk of chronic disease, as there is a significant ecosystem of social-environmental factors that are less proximal to the physiologic stress response but of equal importance. To address health disparities, new policies must be enacted to alleviate upstream determinants that create an inequitable burden of stress, which will, in turn, improve health outcomes.

## Supporting information

S1 File Supplementary methods. This is the supplementary methods that provides deeper explanation of the methodological approach taken by the authors.
(PDF)

S1 Table Biomarkers table. This supplementary table shows the biomarkers used in the algorithm, the cutpoints for their respective quartiles, and the structured codes used to define these measurements.
(PDF)

S2 Table RXCUI medication table. This shows RXCUI codes for medications used to identify dysregulation in the cardiovascular and endocrine systems.
(PDF)

S3 Table Inverse Probability of Selection Weighting Descriptive Statistics table. This shows how both the weighted and unweighted distribution of the cohort.
(PDF)

S4 Table Supplemental regression analysis. This shows the regression analysis presented in the main manuscript when biomarkers are restricted to no more than 2 years apart.
(PDF)
