# Supplementary material for: Perceived stress and allostatic load: Results from the All of Us Research Program
Source: PLoS One. 2025 Aug 8;20(8):e0330106. doi: 10.1371/journal.pone.0330106 (PMC12334008; doi:10.1371/journal.pone.0330106)
Supplement: S1 File — This is the supplementary methods that provides deeper explanation of the methodological approach taken by the authors. (PDF) [file pone.0330106.s001.pdf]

S1 Methods:

We utilized inverse probability weighting (IPW) to address the non-random sampling present in the EHR for the availability of these biomarkers[41]. This correction is intended to mitigate bias that arises in estimating the association between stress and AL due to factors that can influence selection into the EHR. We fit a logistic regression model for the availability of the AL score among those individuals potentially eligible for our analysis, accounting for age, race and ethnicity, education, employment, sex at birth, and where the participant primarily received care (doctor's office, emergency room, urgent care, multiple places, and other). We used the predicted probabilities from this model to estimate inverse-probability weights for each individual, which were ultimately applied to the outcome model. Weights were truncated at the 1st and 99th percentiles to prevent the undue influence of outliers. **Supplemental Table 3** contains the distributions of the selection cohort, unweighted final cohort, and weighted final cohort.
