# Supplementary material for: Perceived stress and allostatic load: Results from the All of Us Research Program
Source: PLoS One. 2025 Aug 8;20(8):e0330106. doi: 10.1371/journal.pone.0330106 (PMC12334008; doi:10.1371/journal.pone.0330106)
Supplement: S1 Table — This supplementary table shows the biomarkers used in the algorithm, the cutpoints for their respective quartiles, and the structured codes used to define these measurements. (PDF) [file pone.0330106.s002.pdf]

S1 Table. Components of allostatic load algorithm, including biomarkers, medication usage, and asthma diagnosis

System	Measurement	Source	Critical Value	LOINC/ICD
Cardiovascular	Systolic Blood Pressure	Physical Measurement	≥133 mmHg	8480-6, 1751-7
	Diastolic Blood Pressure	Physical Measurement	≥82 mmHg	8462-4
	Heart Rate	Physical Measurement	≥80 bpm	8867-4
Lipid Metabolism	HDL Cholesterol	EHR	Female ≤47.00 mg/dL;<br>Male ≤ 39.00 mg/dL;<br>Other ≤ 45.00 mg/dL	2085-9
	Total Cholesterol	EHR	≥211 mg/dL	2093-3
	Triglycerides	EHR	≥148 mg/dL	2571-8
Renal Function	Creatinine	EHR	Female ≥0.88 mg/dL;<br>Male ≥1.10 mg/dL;<br>Other ≥0.96 mg/dL	2160-0
Glucose Metabolism	Glucose	EHR	≥107 mg/dL	2345-7
Immune	Albumin	EHR	≤39.00 g/L	1751-7
	White Blood Cell Count	EHR	≥8.5 #/volume	6690-2
	Asthma Diagnosis	EHR/ Self Report	N/A	**ICD9 Codes:** 493.9, 493.2, 493.0, 493.1, 493.8<br>**ICD10 Codes:** J45.2, J45.3, J45.4, J45.9, J45.99, J45.5, J45.90<br>Self Report (Survey)
Anthropometric	Waist to Hip Ratio	Physical Measurement	Female ≥0.89;<br>Male ≥0.99;<br>Other ≥ 0.93	62409-8, 56086-2
