# Supplementary material for: Perceived stress and allostatic load: Results from the All of Us Research Program
Source: PLoS One. 2025 Aug 8;20(8):e0330106. doi: 10.1371/journal.pone.0330106 (PMC12334008; doi:10.1371/journal.pone.0330106)
Supplement: S2 Table — This shows RXCUI codes for medications used to identify dysregulation in the cardiovascular and endocrine systems. (PDF) [file pone.0330106.s003.pdf]

S2 Table . RXCUI codes for medications used to identify dysregulation in the cardiovascular and endocrine systems.

Condition	RXCUI Codes
Antihypertensives	358274, 1523, 75207, 2599, 3118, 62174, 3327, 3409, 49276, 5036, 40114, 5470, 5784, 6131, 1442132, 6673, 235829, 6876, 266604, 6984, 30257, 7476, 7930, 33717, 8629, 9259, 9260, 55679, 1439816, 10828, 39230, 644, 1369, 1808, 2396, 2409, 62349, 4603, 5487, 5495, 5764, 6628, 6916, 8565, 38413, 10763
Diuretics	644, 1369, 1808, 2396, 2409, 62349, 4603, 5487, 5495, 5764, 6628, 6916, 8565, 38413, 10763
Drugs used for Diabetes	16681, 173, 1534763, 1368001, 1368384, 1368402, 18880, 1373458, 1545149, 2068, 2404, 1488564, 1486436, 2117292, 1727500, 1551291, 1545653, 1598392, 2281864, 1664314, 1992672, 1992684, 1992825, 60548, 102846, 4816, 25789, 647235, 606253, 4821, 25793, 4815, 102848, 26344, 51428, 1670007, 1727493, 139825, 274783, 1858994, 400008, 86009, 253182, 221109, 1100699, 1243019, 475968, 1440051, 6809, 607999, 802646, 614348, 1043562, 729717, 30009, 274332, 8129, 33738, 139953, 73044, 84108, 857974, 1991302, 1189803, 10633, 10635, 38386, 72610, 596554
Lipid Modifying Agents	16817, 1659152, 404773, 83367, 1422085, 2282403, 2283229, 1525, 596723, 2447, 1433887, 21149, 2594, 141626, 2685, 3292, 24609, 2478335, 1665684, 341248, 2535748, 484211, 8703, 1430892, 41127, 4719, 2588243, 1364479, 6472, 352387, 1367839, 7393, 104486, 7414, 861634, 69440, 42463, 8699, 301542, 36567, 38248
