# Supplementary material for: Perceived stress and allostatic load: Results from the All of Us Research Program
Source: PLoS One. 2025 Aug 8;20(8):e0330106. doi: 10.1371/journal.pone.0330106 (PMC12334008; doi:10.1371/journal.pone.0330106)
Supplement: S3 Table — This shows how both the weighted and unweighted distribution of the cohort. (PDF) [file pone.0330106.s004.pdf]

**S3 Table.** Characteristics of participants who meet all inclusion criteria except having all measured allostatic load components among whom inverse probability weights were estimated (n=35,579), the distribution of those meet all inclusion criteria (n=7,415), and the distribution after the inverse probability weights (IPW) for selection are applied.

| **Characteristics** | **IPW Cohort** | **Unweighted Analysis Cohort** | **Weighted Analysis Cohort** |
|---|---|---|---|
| **Age Group** | | | |
| Under 40 | 33.30% | 22.50% | 33.20% |
| 40 to 54 | 32.80% | 35.50% | 32.80% |
| 55 and Above | 33.90% | 42.00% | 34.00% |
| **Sex at Birth** | | | |
| Female | 69.40% | 70.30% | 69.70% |
| Male | 28.40% | 27.60% | 28.00% |
| Unknown | 2.20% | 2.20% | 2.30% |
| **Race and Ethnicity** | | | |
| NHW | 70.30% | 73.50% | 70.40% |
| NHB | 8.70% | 9.90% | 8.60% |
| Hispanic | 11.70% | 8.80% | 11.40% |
| Other | 5.90% | 4.40% | 6.00% |
| Unknown | 3.50% | 3.30% | 3.60% |
| | | | |
| Private | 3.90% | 68.00% | 68.60% |
| Medicare | 68.50% | 5.20% | 4.20% |
| Medicaid | 15.40% | 16.50% | 16.50% |
| VA/Military | 3.90% | 2.80% | 2.70% |
| None | 8.30% | 7.60% | 8.00% |
| | | | |
| Domestic Partner | 59.70% | 59.90% | 58.20% |
| No Longer Married | 15.40% | 17.90% | 15.80% |
| Never Married | 22.20% | 19.80% | 23.30% |
| Unknown | 2.70% | 2.50% | 2.80% |
| | | | |
| Employed | 63.70% | 62.10% | 63.50% |
| Retired | 7.50% | 8.60% | 7.50% |
| Unable | 10.00% | 12.80% | 10.40% |
| Unemployed | 15.90% | 14.00% | 15.60% |
| Unknown | 2.90% | 2.70% | 2.90% |
| **Education** | | | |

| | | | |
|---|---|---|---|
| HS or Less | 12.10% | 12.70% | 11.80% |
| Some College | 24.30% | 23.40% | 24.50% |
| College | 31.00% | 29.60% | 31.20% |
| Advanced | 30.00% | 31.90% | 29.90% |
| Unknown | 2.70% | 2.50% | 2.70% |
| **Birthplace** | | | |
| USA | 87.00% | 88.00% | 86.80% |
| Outside USA | 10.90% | 10.10% | 11.10% |
| Unknown | 2.10% | 1.90% | 2.10% |
| | | | |
| <200% | 25.20% | 25.10% | 24.90% |
| 200-400% | 20.40% | 19.40% | 20.40% |
| >400% | 43.10% | 44.80% | 43.40% |
| Unknown | 11.30% | 10.70% | 11.40% |
| **Loneliness Quartile** | | | |
| Quartile 1 | 30.70% | 30.40% | 29.60% |
| Quartile 2 | 23.20% | 22.40% | 22.80% |
| Quartile 3 | 25.30% | 25.30% | 25.40% |
| Quartile 4 | 20.80% | 21.90% | 22.20% |
| **Everyday Discrimination Quartile** | | | |
| Quartile 1 | 26.10% | 26.40% | 25.60% |
| Quartile 2 | 25.20% | 25.10% | 24.60% |
| Quartile 3 | 23.90% | 24.20% | 23.70% |
| Quartile 4 | 24.80% | 24.30% | 26.00% |
| | | | |
| Secure | 88.80% | 88.20% | 88.10% |
| Insecure | 11.20% | 11.80% | 11.90% |
| **AUDIT C** | | | |
| Not Hazardous | 56.40% | 58.30% | 56.50% |
| Hazardous | 36.30% | 34.50% | 35.50% |
| Unknown | 7.30% | 7.20% | 8.00% |
| **Vaping History** | | | |
| No | 82.00% | 85.00% | 82.20% |
| Yes | 17.50% | 14.50% | 17.20% |
| Unknown | 0.50% | 0.60% | 0.60% |
| **Smoking History** | | | |

| | | | |
|---|---|---|---|
| Never Smoker | 67.90% | 65.10% | 67.10% |
| Current | 8.30% | 9.00% | 8.70% |
| Former smoker | 22.30% | 24.30% | 22.70% |
| Skip | 1.60% | 1.50% | 1.60% |
| | | | |
| Quartile 1 | 25.70% | 26.00% | 24.40% |
| Quartile 2 | 32.90% | 33.30% | 33.50% |
| Quartile 3 | 21.90% | 21.80% | 21.90% |
| Quartile 4 | 19.60% | 18.80% | 20.30% |
| **Neighborhood Disorder Quartile** | | | |
| Quartile 1 | 27.30% | 27.70% | 26.60% |
| Quartile 2 | 26.00% | 26.10% | 25.80% |
| Quartile 3 | 23.30% | 23.30% | 23.60% |
| Quartile 4 | 23.40% | 22.90% | 24.00% |
