# Supplementary material for: Perceived stress and allostatic load: Results from the All of Us Research Program
Source: PLoS One. 2025 Aug 8;20(8):e0330106. doi: 10.1371/journal.pone.0330106 (PMC12334008; doi:10.1371/journal.pone.0330106)
Supplement: S4 Table — This shows the regression analysis presented in the main manuscript when biomarkers are restricted to no more than 2 years apart. (PDF) [file pone.0330106.s005.pdf]

S4 Table. Weight rated odds ratios (OR) and 95% confidence intervals (95% CI) for the association between perceived stress and high allostatic load restricting to 6,129 individuals with all allostatic load components measured in a 2-year time window.

	**Model 1**		**Model 2**		**Model 3**		**Model 4**	
**Stress**	**OR**[1]	**95% CI**[1]	**OR**[1]	**95% CI**[1]	**OR**[1]	**95% CI**[1]	**OR**[1]	**95% CI**[1]
Low	—	—	—	—	—	—	—	—
Moderate	1.23	1.08, 1.40	1.07	0.93, 1.23	1.02	0.87, 1.18	1.03	0.89, 1.20
High	2.20	1.75, 2.75	1.48	1.16, 1.89	1.34	1.02, 1.76	1.36	1.03, 1.79

Model 1 incorporates inverse probability of selection weights and includes age, sex at birth
Model 2 includes for all variables in Model 1 plus education, race and ethnicity, health insurance, nativity, employment, poverty-to-income ratio group, and marital status
Model 3 includes for all variables in Model 2 plus neighborhood disorder scale, neighborhood social cohesion scale, UCLA-Loneliness scale, everyday discrimination scale, and food security scale
Model 4 includes for all variables in Model 3 plus smoking, alcohol consumption, and electronic cigarette use
